# Chemotherapy-Mediated Purging Before Testicular Organoid Generation Significantly Reduces Cancer Cells While Not Significantly Reducing Germ Cells

**DOI:** 10.3390/ijms27177924

**Published:** 2026-09-05

**Authors:** Sven De Windt, Dhoha Kourta, Christine Wyns

**Affiliations:** 1 Pôle de Recherche en Physiopathologie de la Reproduction, Institut de Recherche Expérimentale et Clinique, Université Catholique de Louvain, 1200 Brussels, Belgium; sven.dewindt@uclouvain.be (S.D.W.);; 2 Department of Gynecology-Andrology, Cliniques Universitaires Saint-Luc, 1200 Brussels, Belgium

**Keywords:** testicular organoid, immature testicular tissue, spermatogonial stem cells, fertility restoration, chemotherapy, childhood cancer, artificial testis, fertility preservation, 3D culture, cancer cell decontamination

## Abstract

In total, 37–39% of prepubertal patients present with cancer cells in their testes at the time of diagnosing a hematologic malignancy, and elimination of cancer cells from immature testicular tissue (ITT) remains unsuccessful. Treating testicular single-cell suspensions (TCSs) with chemotherapy before generating testicular organoids (TOs) for in vivo maturation after transplantation could be considered to safely restore fertility with cryo-stored ITT. Five different enzymatic digestion protocols (E1 to E5) were compared but did not show significant differences. For subsequent experiments, E3 using 1.4 mg/mL DNase I and 0.5 mg/mL Liberase was chosen based on the highest recovery of viable testicular cells per mg of tissue and lowest enzymatic concentrations. TCSs were contaminated with a human leukemia HL-60 cell line at concentrations of 4 or 10% and exposed for 24 h to cisplatin (1 µM or 3 µM) or carboplatin (10 µM or 30 µM) prior to TO generation and compared to controls. After eighteen days, hematoxylin and eosin staining showed the development of TOs with forming seminiferous tubules (STs) and interstitium in all conditions. Chemotherapy-treated TOs showed altered germ cell (GC) (DDX4^+^) and somatic (ACTA2^+^, CYP11A1^+^) cell-type-specific location. CD43^+^ HL-60 cells were located outside forming STs in untreated TOs, while in chemotherapy-treated TOs, HL-60 cells were found inside and outside STs. Cancer cell numbers were significantly reduced in 30 µM carboplatin, showing a 3.7-fold reduction, whereas 10 µM carboplatin, 1 µM and 3 µM cisplatin did not demonstrate a statistically significant reduction. DDX4^+^ GC numbers were not statistically reduced after cisplatin or carboplatin exposure. Chemotherapy did not significantly reduce somatic cell numbers. As substantial residual cancer cell contamination remained, it is unlikely that short-term cisplatin/carboplatin treatment as a stand-alone purging strategy will achieve adequate decontamination for a safe fertility restoration approach. Future research should focus on exploring other chemotherapeutic drugs and/or combinations and elucidating the mechanism of chemotherapy-induced altered TO generation.

## 1. Introduction

Despite advanced pediatric cancer therapies and increasing survival rates, now over 80% [1,2], gonadotoxicity leading to infertility still affects 46% of the patients [3]. For adolescent boys facing gonadotoxic therapy, the possibility of parenthood is generally preserved as they can benefit from semen freezing prior to chemotherapy. For prepubertal boys, whose testes do not contain differentiated germ cells (GCs), this is not an option.

In today’s childhood hematological cancer care pathways, immature testicular tissue (ITT) is often cryo-stored for future fertility restoration as part of fertility preservation programs proposed worldwide [4,5]. Cryo-storage of patients’ ITT, containing spermatogonial stem cells (SSCs), is indeed the only experimental fertility preservation option—ethically accepted—to offer them a chance of fathering their own genetic child [4,5,6]. Despite the urgent need, as some of the patients have reached the age to conceive, neither in vitro maturation nor SSCs nor ITT transplantation has led to fertilizing spermatozoa so far. In non-human primates, autotransplantation appears to be the most promising technique with the development of mature sperm and resulting offspring [7]. However, a major concern is the possibility of cancer cell contamination of cryo-stored ITT, as mean contamination rates in pre- and peripubertal boys diagnosed with acute lymphoblastic leukemia or lymphoma indicated 37–39% of the ITT to contain cancer cells [8,9]. Based on animal studies showing that as few as 20 leukemic cells can induce cancer relapse in rats [10] and cancer cells can survive cryopreservation with leukemia relapse in all grafted animals [11], autotransplantation of ITT from patients with hematologic or metastatic malignancies is not recommended and could only be considered if the risk is negligible after a thorough multi-collaborative assessment. Hence, this highlights the need to explore options to obtain cancer-free ITT fragments or testicular single-cell suspensions (TCSs) for safe transplantation. Autotransplantation of properly selected spermatogonia, including SSCs, into the original testicular niche is an option based on preimplantation embryos’ development in non-human primates [12]. Furthermore, selection of human testicular cells allowing a high yield of spermatogonia, including the SSCs, which is essential for niche colonization, is challenging [13,14]. Currently, experimental cancer decontamination strategies for human testicular tissue are mainly based on cell sorting [15,16] or long-term culture [17] (for review, see [8]). FACS/MACS-based cell sorting strategies generally demonstrated disappointing results with either remaining cancer cells detectable after sorting or tumor formation after transplantation of selective putative human SSCs [15,16]. One study reported that there was no tumor formation after transplantation of the putative SSC fraction obtained after multiparametric FACS sorting with EpCAM^+^/HLA-ABC^−^/CD49e^−^ of a MOLT-4-contaminated cell suspension. This putative SSC fraction resulted in a 7.5-fold enrichment of spermatogonia with 98.8–99.8% purity and a 12-fold increase in colonization activity. Furthermore, xenotransplantation of the sorted fraction did not result in tumor development. However, as transplantation of pure MOLT-4 cancer cells resulted in only 23% and 55% of animals developing tumors after intratubular and interstitial transplantation, respectively, this outcome questions the tumor formation capacity of this cancer cell line and thus still the potential of cell sorting methods to achieve a cancer cell-free suspension for safe SSC transplantation in a clinical setting [18].

Creation of human testicular organoids (TOs) with various aims, including the study of drug toxicity and assessment of chemotherapeutic drugs’ testicular gonadotoxicity, has been reported [19,20,21,22]. Based on this knowledge, formation of a transplantable TO free of cancer cells using a TCS pre-treated with chemotherapeutic drugs could be an innovative application in the field of fertility preservation and restoration.

The literature reveals a plethora of protocols for obtaining TCSs using different enzymes, incubation times and conditions, highlighting the importance of maximizing cellular yield and viability (for review, see [23]). This is especially relevant for fertility restoration applications and even more important when chemotherapeutic drugs are used to eradicate cancer cells from cryopreserved tissue or cells as exposure to chemotherapy agents such as cisplatin and carboplatin could further decrease GC viability [24]. In addition, delayed postexposure effects have also been reported. Indeed, 24 h exposure to cisplatin at a low dose (1.67 µM) did not lead to loss of fetal GC numbers 24 h post-exposure, while 72 h, 96 h and 240 h post-exposure assessments revealed a significant reduction in GC numbers (28%, 44% and 67% decrease, respectively) [25]. For low-dose (5 µg/mL) carboplatin, no significant reduction in the number of GCs was observed 72 h post-exposure, but 240 h post-exposure, a 58% reduction in GC numbers was found [25]. Dose-dependent in vitro gonadotoxicity has been described in animal models [26], but dose effects on human testicular cells have not been fully explored. Although no statistically significant difference in fetal GC numbers was demonstrated between low- (1.67 µM) and high-dose (3.33 µM) exposure to cisplatin [25], the effects of higher doses that may be relevant to purge cancer cells from testicular tissue or cells are unknown.

Therefore, the objectives of this study were to (1) identify an enzymatic tissue digestion protocol allowing high viable yield of testicular cells by comparing four original protocols to a protocol based on the most commonly used enzymes, (2) determine the efficacy of cancer cell decontamination of TCSs using two different clinically relevant concentrations of cisplatin (1 µM or 3 µM) or carboplatin (10 µM or 30 µM) [27], and (3) investigate the influence of chemotherapy on TO generation and survival of testicular cells, in particular the scarce GCs, during an eighteen-day period after chemotherapy exposure. As limited amounts of human ITT are available for research purposes, neonatal piglet ITT was selected. Research has already identified the usability of this model over other non-primate animals. Indeed, neonatal piglets demonstrated an extended prepubertal period compared to rodents: data on reproductive processes in pigs determined the weight/volume ratio of neonatal piglet testes, showing high similarity to prepubertal human testes [13,28]. In addition, it is easy to access castration-derived neonatal testes in large quantities [29], and TOs have already been generated with piglet tissue [30].

## 2. Results

### 2.1. Identification of the Enzymatic Digestion Protocol for Subsequent TO Formation Experiments

Total cell viability and viable cells’ yield per mg of tissue, determined by Trypan blue exclusion tests directly after digestion, are presented in Table 1. Total cell viability was in all cases > 92%. The highest yield normalized per mg of digested tissue was obtained using E3, though differences were not statistically significant. In addition, qualitative observation of the TCSs showed more undigested tissue pieces remaining at the end of the protocols using Dispase (E1–2), and less cell debris in the protocols using Liberase (E3–5). Therefore, as E3 also used the lowest enzymatic concentrations, it was selected for further TO generation and decontamination experiments.

### 2.2. Influence on TO Generation of Cisplatin/Carboplatin-Exposed TCSs and/or Presence of HL-60 Cancer Cells

**Figure 1:**
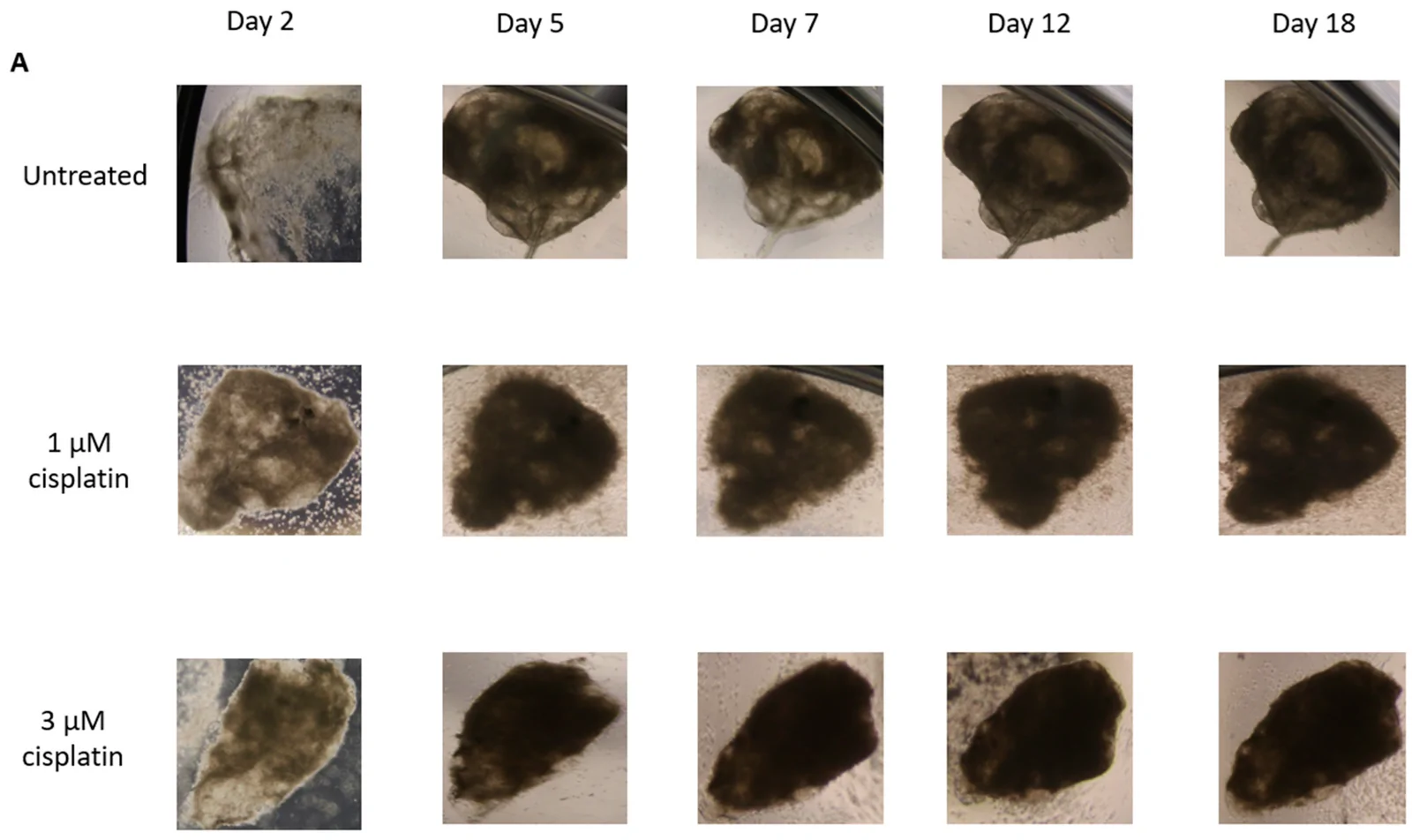

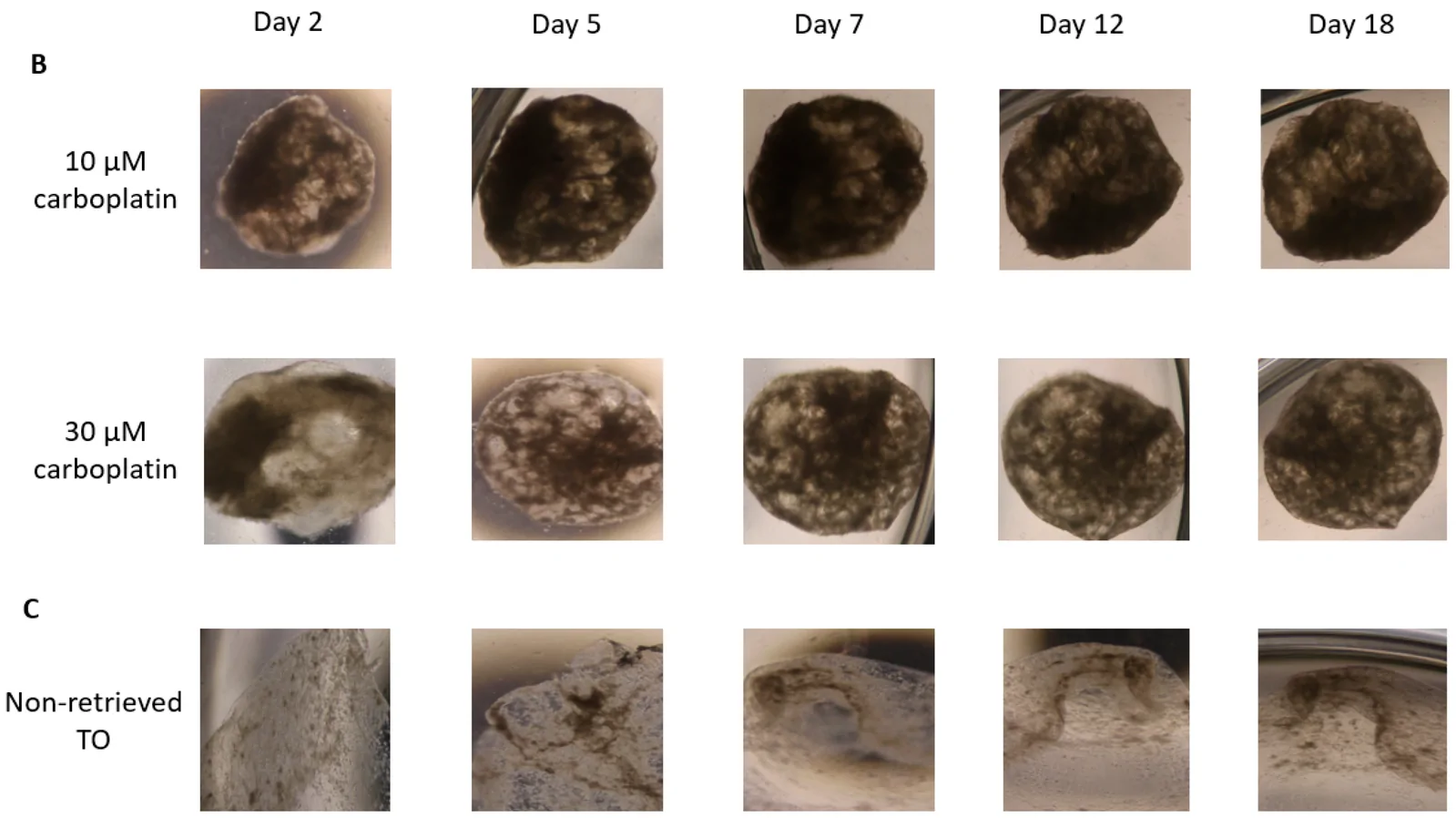
Representative images of the same TO per condition obtained by live imaging during TO generation on day 2, 5, 7, 12 and 18. (**A**) Untreated control, 1 µM and 3 µM cisplatin-treated TCSs; (**B**) 10 µM and 30 µM carboplatin-treated TCSs; (**C**) TCSs without TO generation.

**Figure 2:**
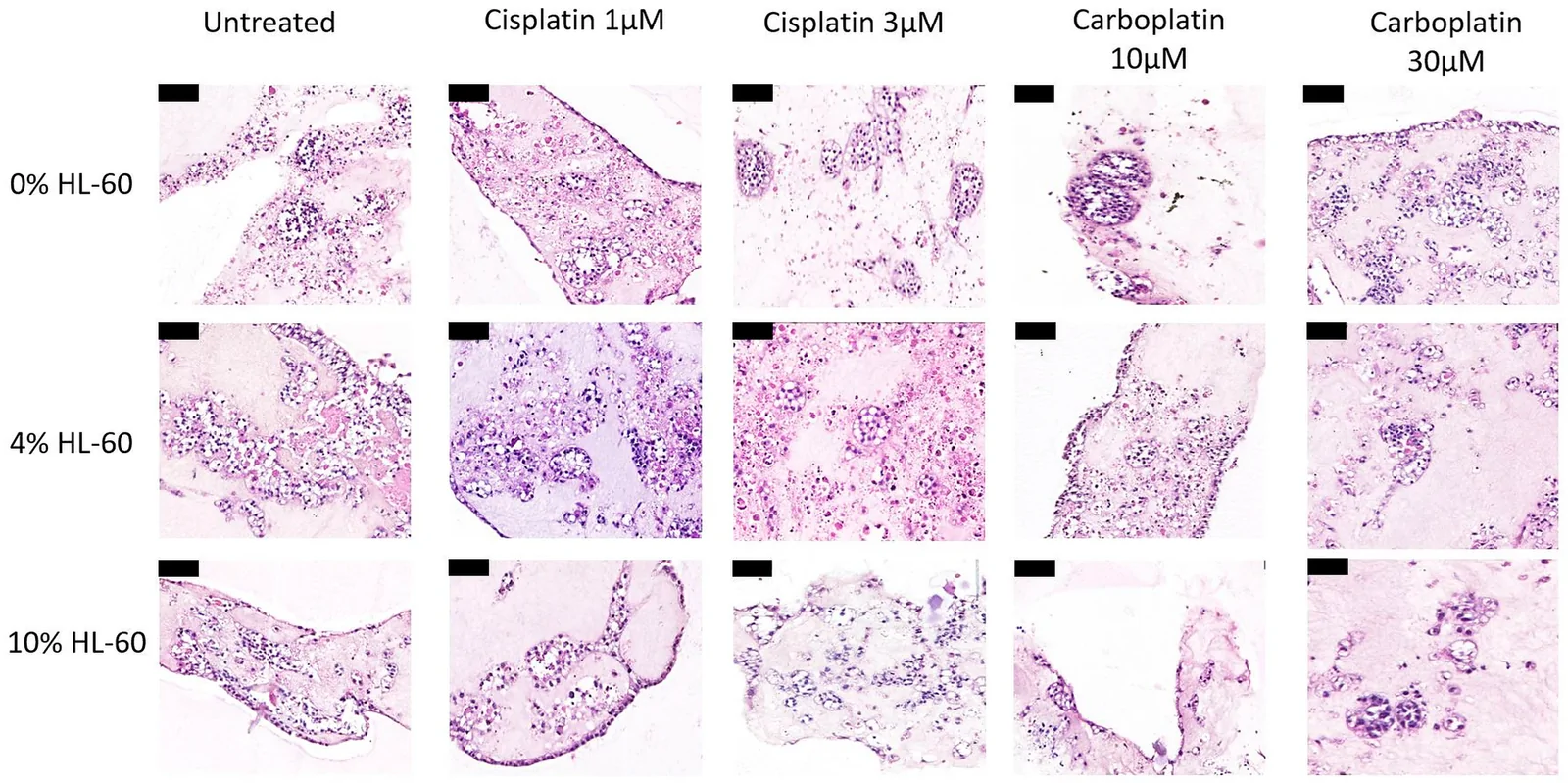
Representative hematoxylin and eosin-stained sections showing TOs with seminiferous tubules (STs) formed at day eighteen. Regions were selected based on the spatial occurrence of STs, which typically appear within a confined area of the hydrogel. Scale bar = 50 µm.

**Figure 3:**
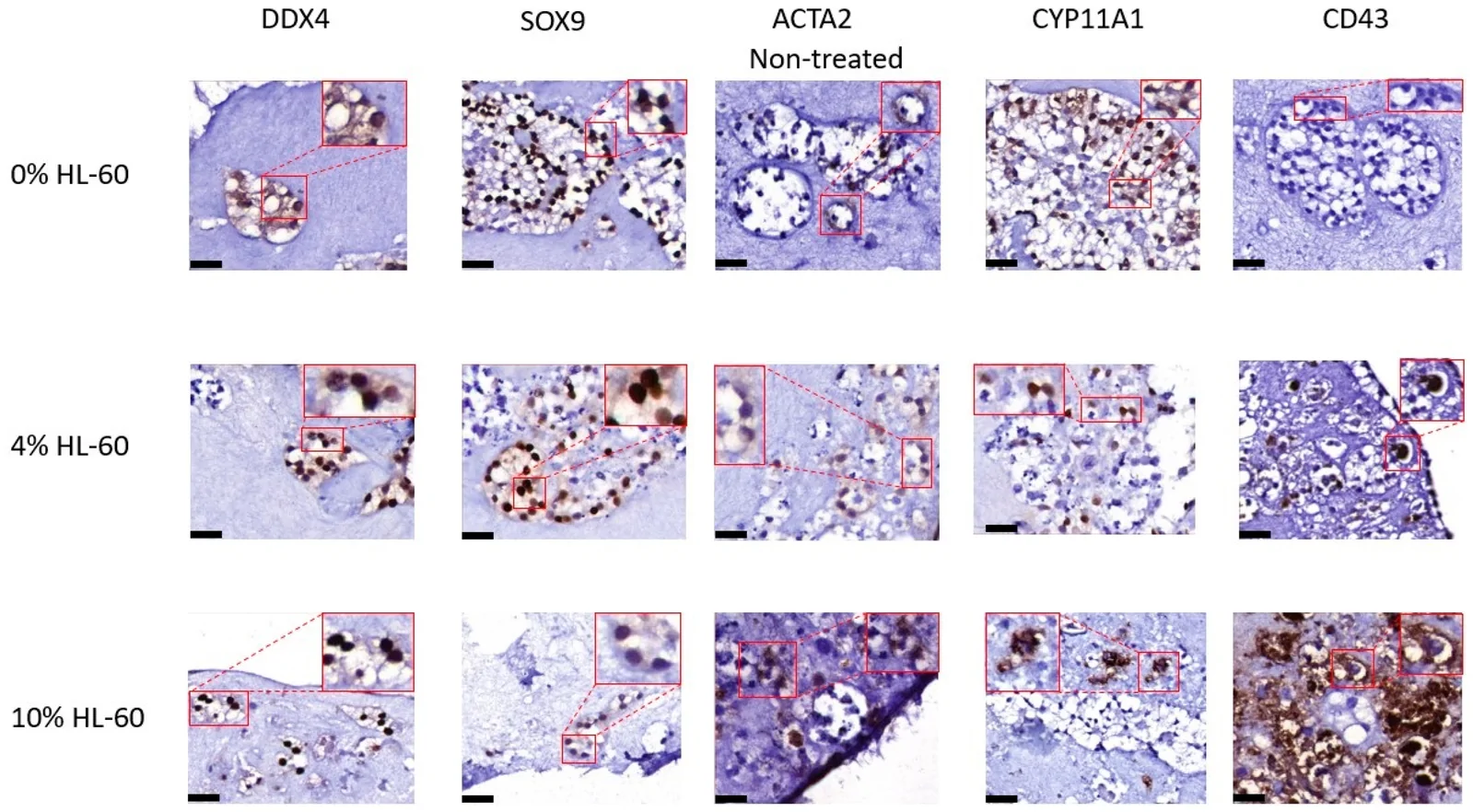
Untreated TCS. Immunohistochemistry demonstrating location of DDX4^+^, SOX9^+^, ACTA2^+^, CYP11A1^+^ and CD43^+^ cell populations in TOs formed with 0%, 4% and 10% HL-60 cells at day eighteen. TOs exhibited cell-type-specific locations resembling the native testicular architecture. Scale bar = 20 µm.

**Figure 4:**
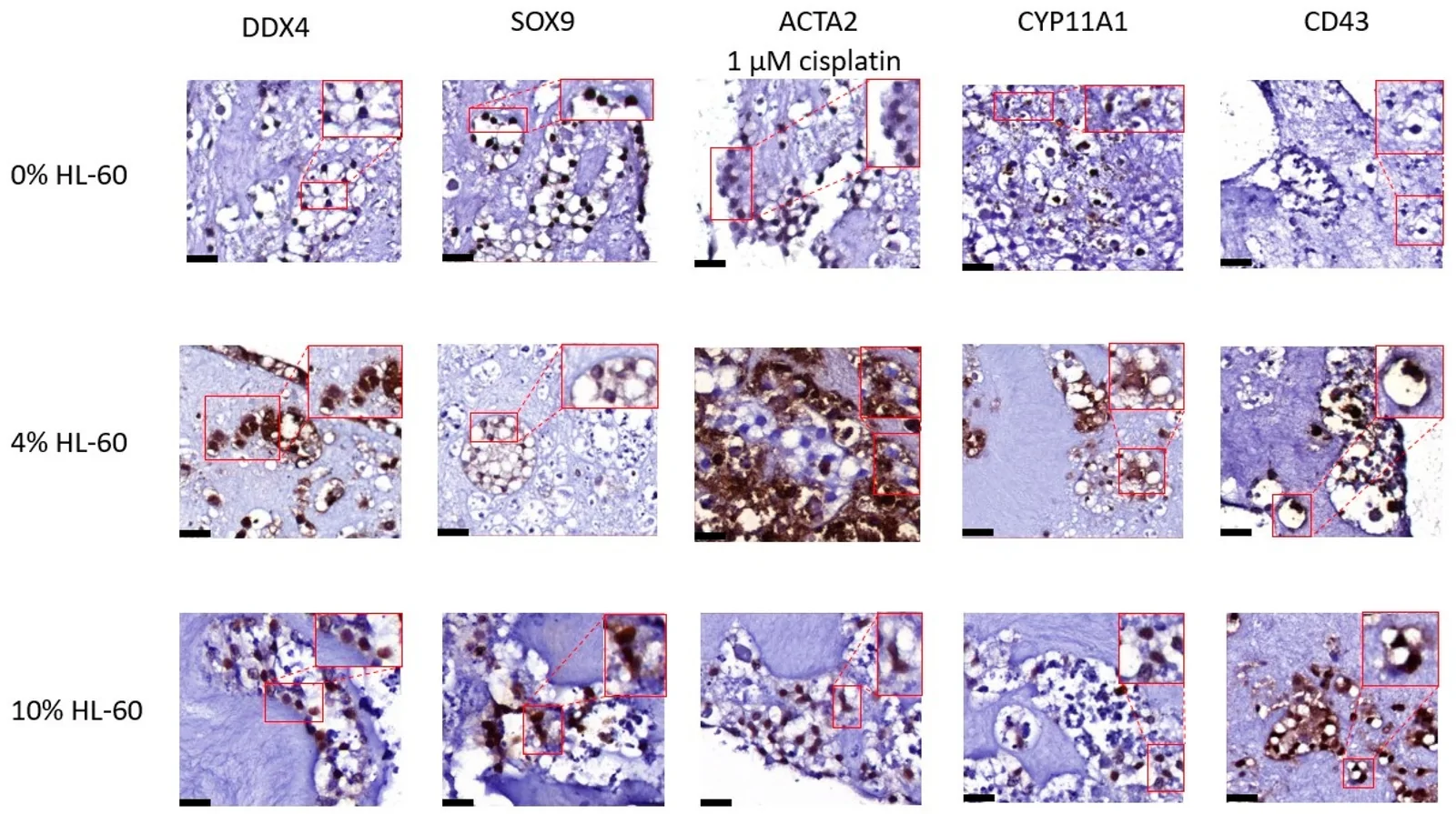
Images of 1 µM cisplatin-treated TCS. Immunohistochemistry demonstrating location of DDX4^+^, SOX9^+^, ACTA2^+^, CYP11A1^+^ and CD43^+^ cell populations in TOs formed with 0%, 4% and 10% HL-60 cells at day eighteen. TOs retained an appropriate location of SOX9^+^ and ACTA2^+^ cells, whereas DDX4^+^ and CYP11A1^+^ cells displayed a modified spatial distribution compared to native tissue. CD43^+^ HL-60 cells were found both inside and outside the forming STs. Scale bar = 20 µm.

**Figure 5:**
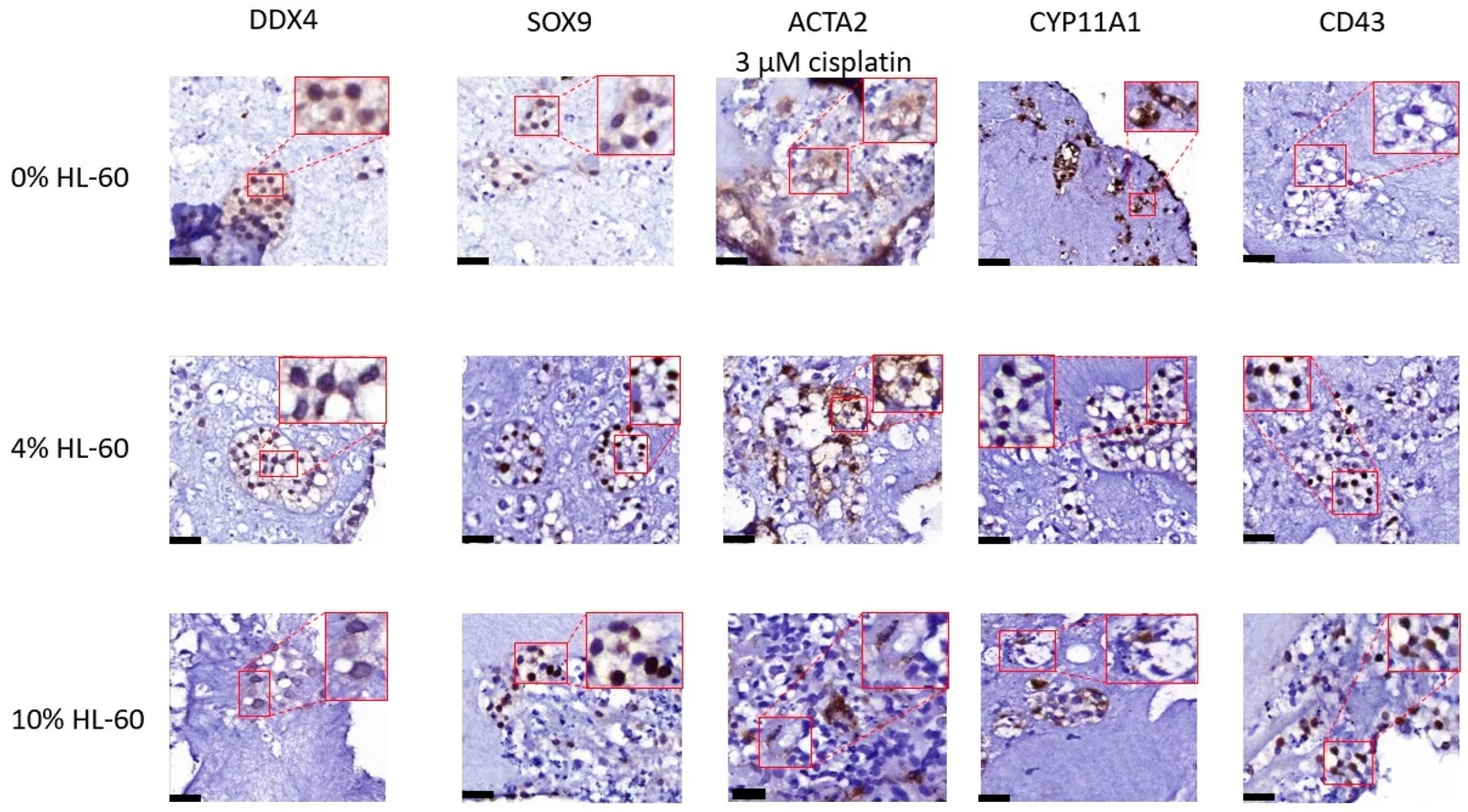
Images of 3 µM cisplatin-treated TCS. Immunohistochemistry demonstrating location of DDX4^+^, SOX9^+^, ACTA2^+^, CYP11A1^+^ and CD43^+^ cell populations in TOs formed with 0%, 4% and 10% HL-60 cells at day eighteen. TOs retained an appropriate location of SOX9^+^ and ACTA2^+^ cells, whereas DDX4^+^ and CYP11A1^+^ cells displayed a modified spatial distribution compared to native tissue. CD43^+^ HL-60 cells were found both inside and outside the forming STs. Scale bar = 20 µm.

**Figure 6:**
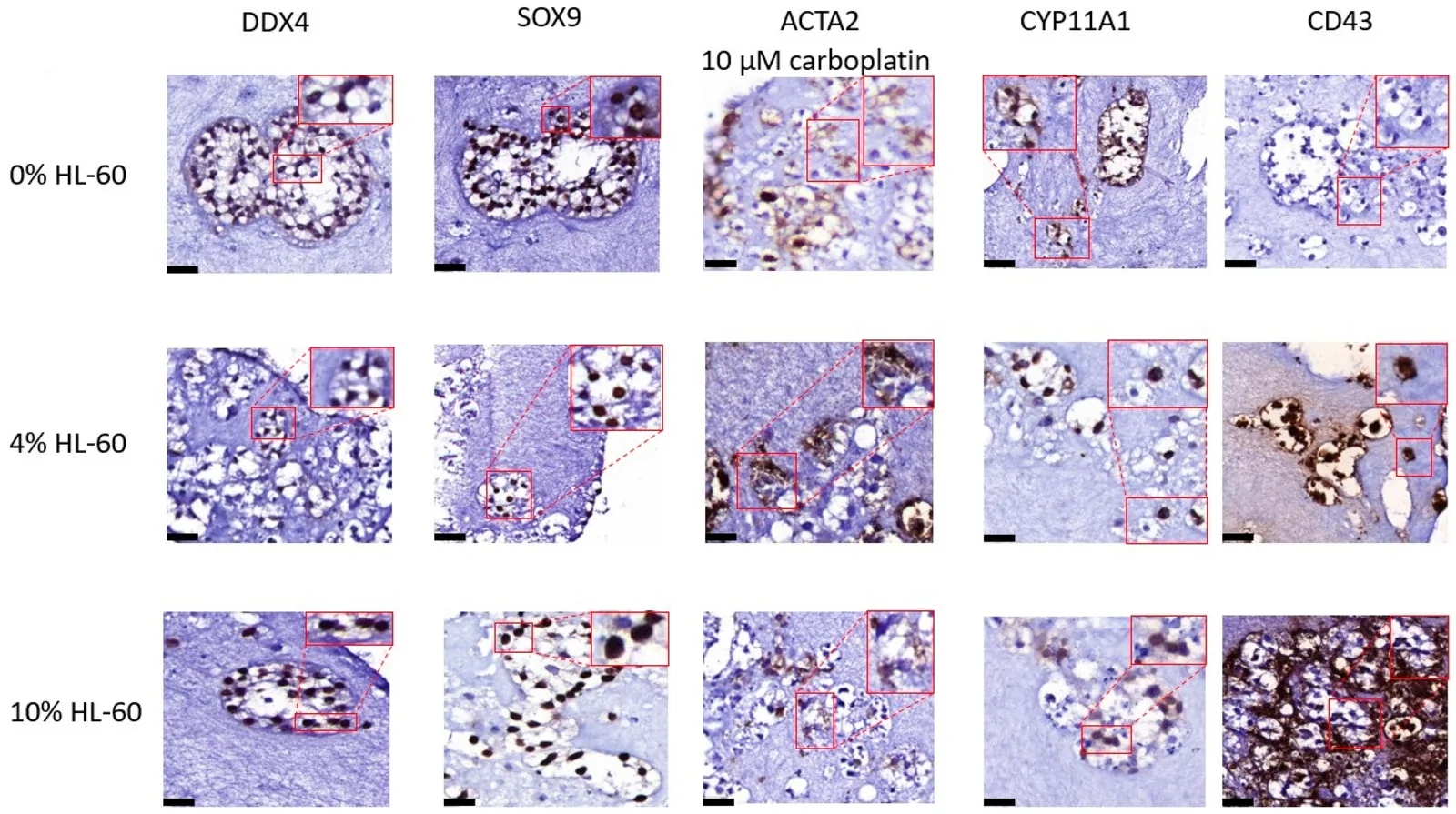
Images of 10 µM carboplatin-treated TCS. Immunohistochemistry demonstrating location of DDX4^+^, SOX9^+^, ACTA2^+^, CYP11A1^+^ and CD43^+^ cell populations in TOs formed with 0%, 4% and 10% HL-60 cells at day eighteen. TOs retained an appropriate location of DDX4^+^, SOX9^+^, and ACTA2^+^ cells, while CYP11A1^+^ cells showed a modified location within the TOs. CD43^+^ HL-60 cells were found both inside and outside the forming STs. Scale bar = 20 µm.

**Figure 7:**
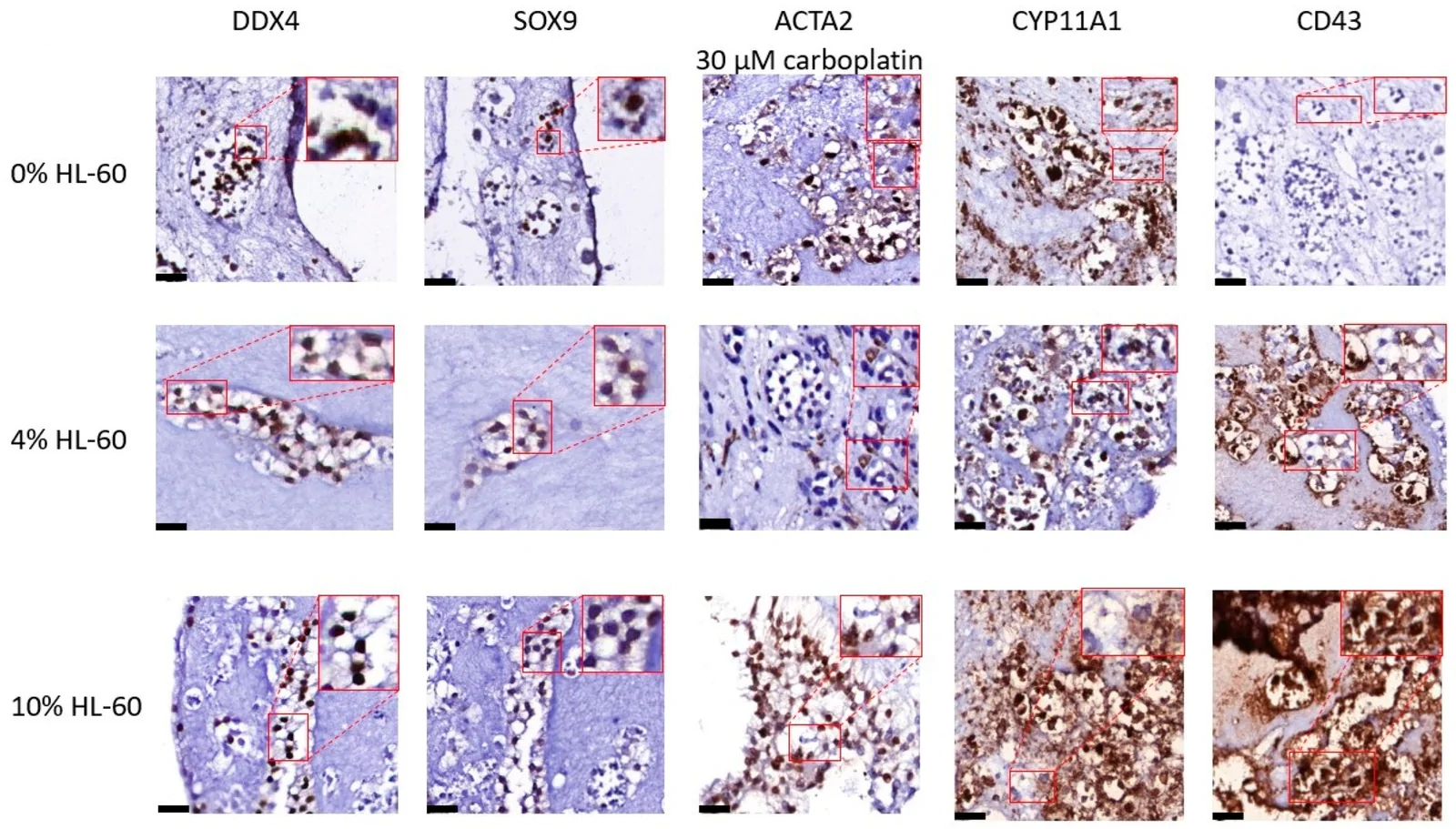
Images of 30 µM carboplatin-treated TCS. Immunohistochemistry demonstrating location of DDX4^+^, SOX9^+^, ACTA2^+^, CYP11A1^+^ and CD43^+^ cell populations in TOs formed with 0%, 4% and 10% HL-60 cells at day eighteen. TOs retained an appropriate location of DDX4^+^, SOX9^+^, and ACTA2^+^ cells, while CYP11A1^+^ cells showed a modified location within the TOs. CD43^+^ HL-60 cells were found both inside and outside the forming STs. Scale bar = 20 µm.

TO generation was visualized in real time by live imaging (Figure 1), allowing the evaluation of growth and density. Only TOs demonstrating a dense structure, indicated by lower light penetration over time, were retrieved for further analyses. This selection criterion led to recovery rates ranging from 40.00 to 93.33% of the total number of TOs per condition (Table 2). Hematoxylin and eosin (H&E) staining performed at day eighteen revealed the formation of seminiferous tubules structures (STs), with varying ST diameters, surrounded by a basement membrane and cells present in the interstitial space in all groups, with Sertoli cells (SCs) stained for SOX9 present inside forming STs mimicking the in vivo architecture (Figure 2, Figure 3, Figure 4, Figure 5, Figure 6 and Figure 7).

#### 2.2.1. Control TO Formation

IHC revealed a testicular cell location similar to the native testicular architecture in the control group (untreated, 0% HL-60) with GCs (DDX4^+^) and SCs (SOX9^+^) inside STs, peritubular myoid cells (PTMCs) (ACTA2^+^) surrounding forming STs, and Leydig cells (LCs) (CYP11A1^+^) outside the STs (Figure 2 and Figure 3).

#### 2.2.2. Chemotherapy-Treated TCSs Without Initial HL-60 Cancer Cell Contamination

Similar to the in vivo situation, SOX9^+^ SCs were located inside forming STs in both chemotherapy-treated TOs regardless of the dose (Figure 2, Figure 4, Figure 5, Figure 6 and Figure 7).

TOs exposed to cisplatin, independently of the dose, showed an altered cell-type-specific location with DDX4^+^ GCs and CYP11A1^+^ LCs found both inside and outside forming STs. In addition, ACTA2^+^ PTMCs were found outside (in close relation to) the forming STs, mimicking the in vivo situation (Figure 2, Figure 4 and Figure 5).

For carboplatin-treated TOs, regardless of the dose, DDX4^+^ GCs were identified inside forming STs, mimicking in vivo testicular cell-type-specific location. ACTA2^+^ PTMCs were found outside forming STs, but widespread in the interstitial space, thus not only in proximity to forming STs. CYP11A1^+^ LCs were found both inside and outside the forming STs (Figure 2, Figure 6 and Figure 7).

#### 2.2.3. Untreated TCSs with 4% and 10% Initial HL-60 Cancer Cell Contamination

IHC revealed the cell-type-specific location to mimic the in vivo architecture. DDX4^+^ GCs and SOX9^+^ SCs were found inside forming STs. CYP11A1^+^ LCs and CD43^+^ HL-60 were identified outside the STs in the examined sections. For ACTA2^+^ PTMCs, the location tended to resemble, but did not closely mimic, the in vivo situation (Figure 2 and Figure 3).

#### 2.2.4. Chemotherapy-Treated TCSs with 4% and 10% Initial HL-60 Cancer Cell Contamination

Cell location assessment revealed identical results for both chemotherapy-treated TOs with and without initial HL-60 cancer cell contamination. Independent of the initial HL-60 cancer cell concentration, cisplatin-treated TOs demonstrated DDX4^+^ GCs and CYP11A1^+^ LCs located both inside and outside forming STs. Furthermore, ACTA2^+^ PTMCs were found outside, though in close relation to the forming STs, mimicking the in vivo situation (Figure 2, Figure 4 and Figure 5). For carboplatin-treated cancer-contaminated TCSs, assessment of cell location showed DDX4^+^ GCs to be found inside forming STs. In addition, ACTA2^+^ PTMCs were found outside forming STs, but were widespread, thus not only in proximity to forming STs. CYP11A1^+^ LCs were found inside and outside forming STs (Figure 2, Figure 6 and Figure 7).

### 2.3. Detection of HL-60 Cells in TOs Derived from Untreated and Chemotherapy-Treated TCSs

Interestingly, TCSs not exposed to chemotherapy demonstrated CD43^+^ HL-60 cancer cells located outside forming STs in the examined sections (Figure 3). On the contrary, TCSs exposed to chemotherapy, independent of dose or initial HL-60 cell contamination, demonstrated CD43^+^ HL-60 cancer cells both inside and outside forming STs (Figure 4, Figure 5, Figure 6 and Figure 7).

### 2.4. HL-60 Cell Decontamination Efficacy of Cisplatin/Carboplatin During TO Generation

**Figure 8:**
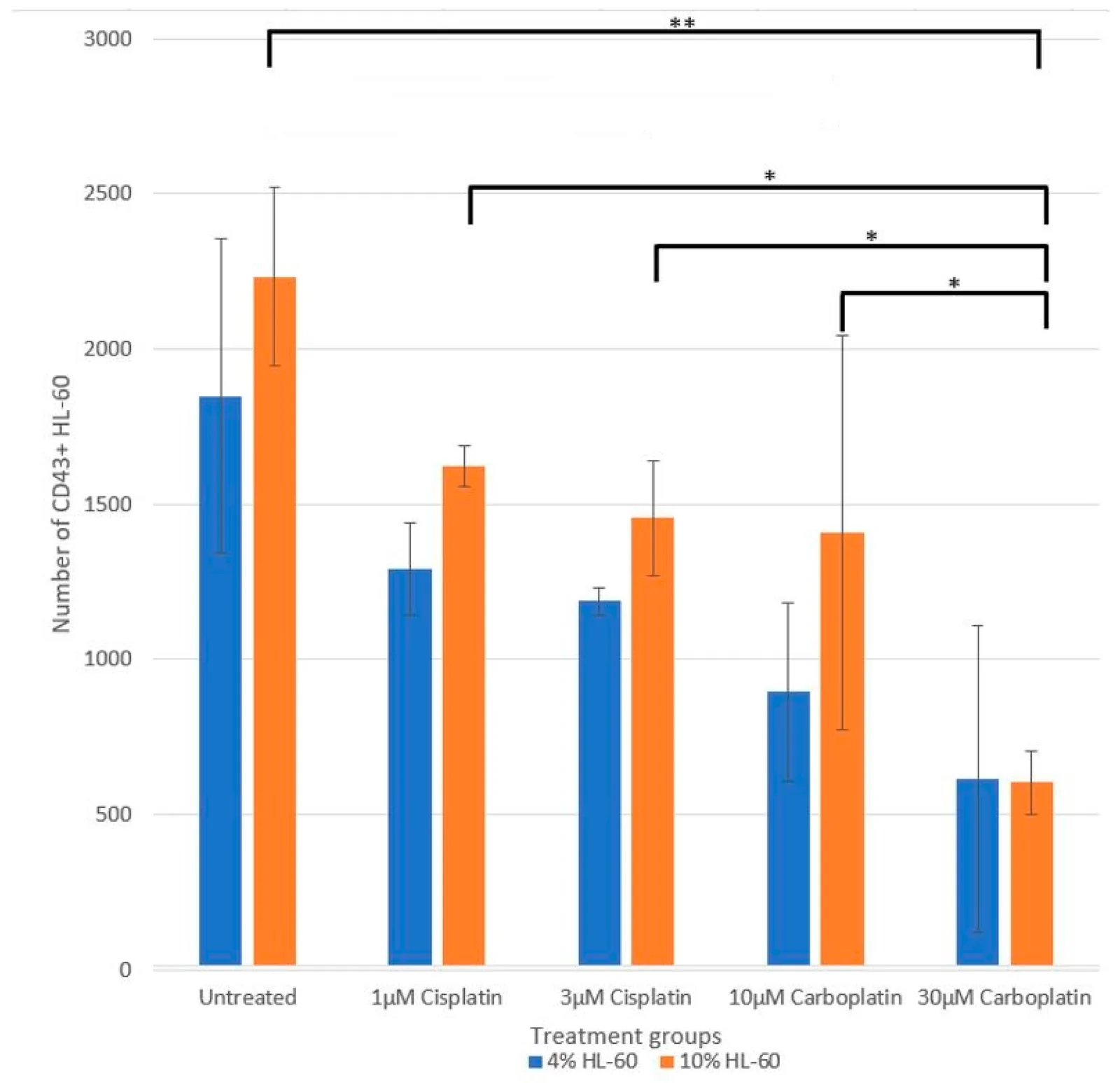
CD43^+^ quantification expressed as cell numbers in TOs formed after TCS exposure to chemotherapy and untreated controls normalized per TO for the 2 different HL-60 contamination rates at day eighteen. A Kruskal–Wallis non-parametric test followed by a post hoc Dunn’s test with Bonferroni correction was used for comparison of HL-60 cancer cell numbers among the different treatment groups. * *p* < 0.05; ** *p* < 0.02.

To verify the decontamination efficacy of both doses of chemotherapeutics, quantification of CD43^+^ cancer cells was performed and recorded as proportions and cell numbers, with data normalized per TO and compared to the untreated TOs (Table 3, Figure 8).

The highest proportions of HL-60 cancer cells were found in the 4% and 10% initial HL-60 groups in untreated TCSs with 36.80 ± 40.93% and 37.41 ± 11.70% CD43^+^ cells, respectively. Cisplatin and carboplatin reduced the proportions of CD43^+^ cells in a dose-dependent manner, though this was not statistically significant. Furthermore, 30 µM carboplatin treatment resulted in the lowest CD43^+^ proportions with 15.51 ± 11.18% and 25.16 ± 3.50% CD43^+^ cells in the 4% and 10% HL-60 cancer cell-contaminated TCSs, respectively.

CD43^+^ HL-60 cell numbers were not statistically different between conditions in the 4% initial HL-60 cancer cell-contaminated TCSs. On the contrary, a significant decrease compared to untreated control TOs was observed when 10% HL-60 initial cancer cell-contaminated TCSs were treated with 30 µM carboplatin. In addition, 30 µM carboplatin in 10% HL-60 groups demonstrated a statistically significant beneficial effect in reducing CD43^+^ HL-60 cells compared to all other chemotherapy treatments (Figure 8). Although the high dose of 30 µM carboplatin did not result in complete cancer cell decontamination, it allowed a 3.7-fold reduction in the number of CD43^+^ HL-60 cancer cells.

### 2.5. Influence of Cisplatin/Carboplatin on TCSs During TO Generation on Testicular Cell Proportions and Numbers

To investigate the influence of both chemotherapeutic drugs and their corresponding doses on testicular cells, a quantification was performed for each testicular cell type, normalized per TO, and compared to the untreated groups (Table 4). No effect of cisplatin or carboplatin exposure on GC numbers could be observed, and GCs were still present after eighteen days in all conditions. In addition, although a dose-dependent trend of decreasing DDX4^+^ GC proportions and numbers was observed when comparing chemotherapy-treated TCSs with untreated controls, this reduction in cell proportion was only significant after 30 µM carboplatin exposure compared to the untreated control (1.52 ± 0.36% and 592.17 ± 30.48 vs. 4.02 ± 0.45% and 1242.17 ± 72.65 DDX4^+^ GCs, respectively). Proportions and cell numbers of somatic cells showed a non-dose-dependent trend of lower SOX9^+^ and ACTA2^+^ in chemotherapy-treated TCSs compared to untreated controls, though not statistically significant. CYP11A1^+^ LC proportions and numbers remained similar when comparing chemotherapy-treated and untreated TCSs.

## 3. Discussion

Despite the urgent need to offer a safe fertility restoration method as some patients have now reached reproductive age and wish to father their own genetic child, still no efficient validated decontamination protocol has been obtained to purge human ITT of cancer cells [8]. Multiple studies have tried to decontaminate prior artificially contaminated TCSs with different cancer cell lines. MOLT-4 cancer cells were used in the study of Dovey et al. (2013) to artificially contaminate the TCS, but demonstrated low proliferative and invasive capacities, as only 23% and 55% of the animals developed cancer after xenotransplanting pure MOLT-4 cancer cells in the tubular and interstitial compartments, respectively [18]. B-cell lymphoblastic leukemia cells were used in the study by Geens et al. (2011), but 5/5 tested TCSs demonstrated cancer cell presence after cell sorting [15]. The same cancer cell line was used by Sadri-Ardekani et al. (2014) to eradicate cancer cells after long-term culture [17]. The method appeared effective in cancer cell decontamination based on the absence of detectable cancer cells by minimal residual disease PCR, but the reproducibility of these results needs further confirmation [17]. While pursuing efforts to achieve TCSs that could allow a safe clinical application with both of these methods, an interesting potential clinical tissue purging approach could consist of digesting prepubertal ITT, eliminating cancer cells from the TCSs with chemotherapy drugs and forming a transplantable TO for further in vivo differentiation of GCs into mature spermatozoa.

In an attempt to develop a protocol able to eliminate cancer cells from TCSs as a first step to achieve a safe fertility restoration strategy, we explored the potential of short-term exposure (24 h) to chemotherapeutic drugs at two clinically relevant doses to eliminate cancer cells from TCSs obtained after ITT enzymatic digestion, and assessed remaining GC numbers at day eighteen. To the best of our knowledge, a study investigating multiple doses of both cisplatin and carboplatin prior to TO generation for its influence on different testicular cell types and presence of cancer cells was never performed. Besides being widely used for oncologic therapies, cisplatin also demonstrated its anti-cancer capacities in vitro by inducing apoptosis in several leukemia cell lines [31,32,33,34]. Similarly, administration of a single dose of carboplatin led to 120-fold higher caspase 3-apoptosis in the HL-60 leukemia cancer cell line compared to untreated HL-60 cell culture [35]. In this study, we selected the commercially available immortalized human acute myelocytic leukemic HL-60 cell line because of its well-known high proliferative potential with a doubling time of 20–45 h and previous demonstration of purging potential in culture with cisplatin [31,36]. As human ITT for research purposes is scarce, neonatal piglet ITT was used, given that the cells’ organization and weight ratio are similar to human prepubertal ITT [13]. Regardless of the treatment and initial HL-60 cancer cell contamination rate, all TOs demonstrated formation of STs and interstitial space based on H&E staining. Cell-type-specific location in TOs was similar to native tissue for untreated groups with or without initial HL-60 cancer cell contamination. For chemotherapy-exposed TCSs, we observed modifications of the testicular cells’ organization in TOs after TCSs’ chemotherapeutic drug exposure, independent of initial HL-60 cancer cell contamination, indicating the chemotherapy to be responsible for altered cell-type-specific location during TO generation. Modification of the cells’ location was different for cisplatin and carboplatin. In particular, DDX4^+^ GCs were observed both inside and outside STs treated with cisplatin. On the contrary, carboplatin treatment demonstrated no influence on DDX4^+^ GC location, as in all HL-60 cancer cell-contaminated groups, the DDX4^+^ GCs were found inside forming STs, likely offering an advantage of using carboplatin for appropriate TO generation free of cancer cells. Among somatic cells, SOX9^+^ SCs and ACTA2^+^ PTMCs showed a similar location to that in native tissue independently of treatment and initial HL-60 cell contamination. However, while ACTA2^+^ PTMCs were located in close proximity to the forming STs in the presence of cancer cells, it is not excluded that based on the absence of a clear continuous lining, cancer cells might have interfered with the organization and structural integrity of the forming STs. Therefore, further confirmation of the TO formation in the presence of cancer cells should be obtained. By contrast, LCs were found both in the interstitium and inside the STs in all chemotherapy-treated TOs, regardless of the initial HL-60 cell concentration. This finding is of particular importance regarding fertility restoration as LCs inside STs would imply that GCs would likely be exposed to a direct effect of testosterone, leading, as already demonstrated in the literature, to GC death [37]. Based on these findings, chemotherapy seems to potentially influence the normal cell signaling necessary to generate TOs that mimic the native testicular architecture. A potential mechanism can be somatic cell dysfunction, as dedifferentiation of SC and LCs after chemotherapy exposure was reported [38]. Furthermore, animal models revealed cisplatin to lead to morphological SC damage, including vacuolation of the cells and elevated levels of oxidative stress [39,40], thus indicating a dysfunction of the SCs that could influence TO generation, as these cells, together with PTMCs, are the primary actors in seminiferous cord formation [41,42]. In the same line, adjacent SCs form the blood–testis barrier, consisting of gap and tight junctions, which will shield differentiating GCs from their own immune system attacks. Our results demonstrated, in the non-treated condition with or without cancer contamination, that cell-type-specific location is similar to the in vivo situation, by contrast with the chemo-treated groups. This leads to the hypothesis that chemotherapy impacts ST generation. In support of this, chemotherapy was demonstrated to influence gap and tight junction intercellular communications, which led to the generation of anti-cancer therapies specifically targeting gap and tight junctions [43,44,45]. In addition, Nishiguchi et al. (2019) demonstrated that targeting claudin-4, a main component of the tight junction, with anti-CLDN4 antibodies may sensitize tumor cells to chemotherapy due to conformational changes [46]. Data also support an impact of cisplatin and carboplatin on gap and tight junctions. Indeed, CX 43, a gap junction and important component of the blood–testis barrier, was found to be a main target of cisplatin [47,48,49]. Furthermore, carboplatin-treated mice demonstrated significantly lower expression of claudin-1 compared to non-treated controls in the small intestine [50]. Based on these observations, we could assume that cisplatin and carboplatin in our study were responsible for impaired junctional complexes between SCs leaving the passage open to other cells such as LCs and HL-60 cancer cells, although no specific verification was performed in this study. Hence, a better option could be to treat the formed TO with chemotherapy rather than the TCS before TO generation.

TO generation was assessed after TCS exposure to chemotherapy to investigate the effect of chemotherapy on testicular cell proportions, numbers and cell-type-specific location.

Assessment of testicular cell proportions and numbers indicates that DDX4^+^ GC proportions and numbers decrease in a dose-dependent manner after chemotherapy exposure. This decrease was not statistically significant except for 30 µM carboplatin exposure that led to decreased GC proportions. However, the presence of remaining GCs and the fact that no statistically significant difference in GC numbers could be observed in TOs eighteen days after drug exposure is an important finding in the context of restoring reproductive potential and leaves some room for further research on chemo-based cancer cell eradication methods. So far, the only study performed on human fetal and prepubertal ITT analyzed GC numbers only up to 240 h post-cisplatin or carboplatin exposure and showed steadily decreasing numbers at 72 h and 96 h [25]. It is likely that after post-exposure effects, recovery of GC numbers occurs from the reserve pool [51] as reported in mice, where 0.25 µg/mL (0.84 µM) and 0.75 µg/mL (2.51 µM) cisplatin exposure for 24 h on prepubertal testicular cells led to a significant decrease in GC numbers after one week of exposure, but recovery at four weeks [52].

Short-term exposure to 30 µM carboplatin significantly decreased the numbers of CD43^+^ HL-60 cancer cells, showing a 3.7-fold reduction compared to untreated controls, but 30 µM carboplatin-exposed TCS-derived TOs still harbored 25.16 ± 3.50% CD43^+^ HL-60 cells. This constitutes a clear limitation of short-term cisplatin/carboplatin treatment to be applied as a stand-alone purging strategy for safe fertility restoration. Interestingly, our results also indicated CD43^+^ HL-60 cancer cells to be located outside forming STs in the untreated TOs. This finding may lead to novel strategies that may lead to safe fertility restoration when ITT is contaminated with cancer cells. If the potential to obtain STs free of cancer cells is further confirmed, they could potentially be recovered by recently developed laser capture microdissection tools, allowing the recovery of areas of interest containing living cells. Indeed, Bennett et al. (2014) demonstrated the successful isolation of a viable alveolar duct region, 200 µm thick, by light microscopy-guided laser cutting [53], which opens the potential to isolate parts of formed STs in our TO model with embedding of ST fragments in an extracellular matrix for downstream applications in the field of fertility preservation.

Overall, our data indicated that 24 h chemotherapy exposure did not significantly impact somatic cell proportions and numbers, important for reconstitution of the SSC niche, SSC renewal and differentiation potential. However, 24 h cisplatin exposure demonstrated reduced development of more advanced GCs such as round and elongated spermatids in prepubertal mice even eight weeks post-exposure [52]. Therefore, future research should focus on investigating the effect of chemotherapy on the functionality, including differentiation of testicular cells, particularly the DDX4^+^ GC, in longer-term TO culture experiments. Research indicates that alkylating agents significantly reduce the numbers of spermatogonia in human ITT when exposed in vivo, an effect that was not observed when carboplatin was administered as the sole chemotherapeutic drug. A commonly used mitotic inhibitor, vincristine, demonstrated additional adverse effects on spermatogonia numbers in human ITT when administered in combination with alkylating agents such as cyclophosphamide [54]. Interestingly, no effect of the combinatory use of cisplatin with other alkylating agents was found regarding the number of spermatogonia per ST in vivo. Although this could be due to the very high toxicity of alkylating agents, it could position cisplatin as a potential candidate for combinatory therapy in future research, being aware that the effect on human ITT is correlated with the cumulative dose administered [24]. Exploration of the potential of other chemotherapeutic drugs and/or combinations, with establishment of the optimal cumulative dose, to eliminate cancer cells in testicular cell preparations could eventually lead to both complete cancer cell decontamination and further elucidation of mechanisms involved in altered TO generation.

Although this study adds knowledge to the existing literature regarding cancer cell decontamination strategies of prepubertal testicular tissue, some limitations must be considered while interpreting these results. TO selection for downstream analyses was based on visual assessment of reduced light penetration by live imaging; thus, in the absence of an objective quantitative assessment, it may be a limiting factor for reproducibility. Results for IHC might be influenced by the region analyzed in the TO in case testicular and cancer cells are not homogeneously spread in the collagen gel. To mitigate this risk, a H&E evaluation was performed on the whole TO by random evaluation of each tenth consecutive slide. Furthermore, high standard deviations were observed in the quantification of cell proportions and absolute cell numbers, likely reflecting heterogeneous dispersion of testicular and HL-60 cells within the collagen gel. Because dispersion efficiency might depend on the initial cell-seeding density, these findings justify evaluating different seeding concentrations to assess spatial homogeneity and potentially achieve results with a reduced variability. In addition, proportions of HL-60 cancer cells in chemotherapy-treated conditions were demonstrated to be higher than the initial contamination rate, but lower than the untreated TOs. Together, these results highlight the robust proliferative behavior of HL-60 cells and indicate that, while chemotherapy reduces their proliferation rate, the effect remains insufficient for complete cancer cell decontamination. Lastly, artificial cancer cell contamination relied on the addition of a cancer cell line to TCSs, which might behave differently from invasive cancer cells in testes in vivo and thus not fully represent the clinical situation and interactions between cancer and testicular cells. Creating a 3D testicular model by injecting cancer cells within the tissue could be a better proxy of the in vivo situation for future research, as it would allow the cancer cells to invade the ITT and potentially behave as they do in vivo.

## 4. Materials and Methods

### 4.1. Experimental Design

**Figure 9:**
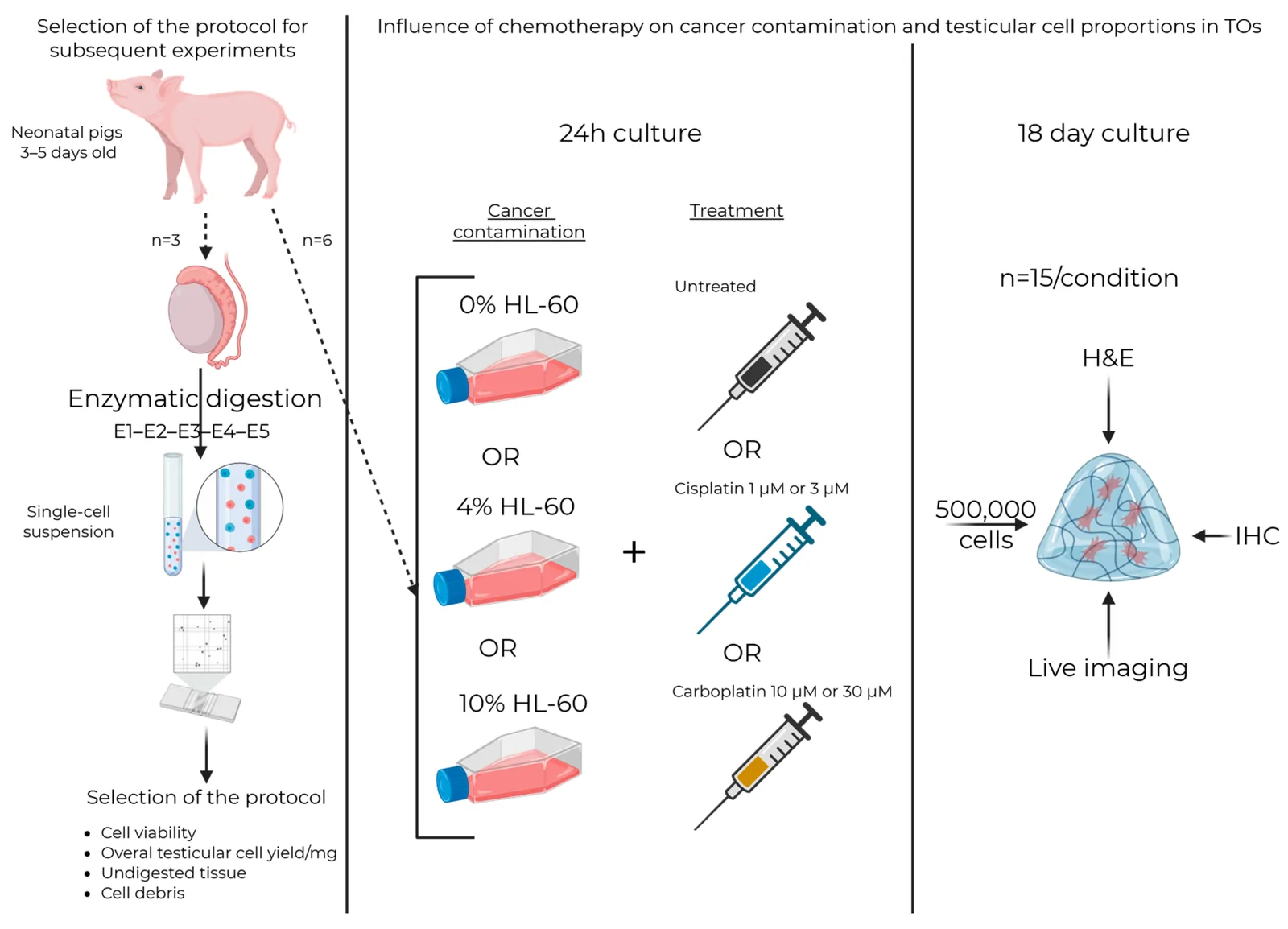
Graphical representation of the study design. HL-60: human leukemia 60 cell line; H&E: hematoxylin and eosin staining; IHC: immunohistochemistry. Created in Biorender. Amorim, C. (2026), https://BioRender.com/tbc3mi0.

This study was conducted as graphically represented in Figure 9. Briefly, neonatal porcine testes (n = 3) were enzymatically digested using five different protocols (E1, E2, E3, E4 or E5, respectively). After digestion, TCSs derived from the same enzymatic digestion protocol were mixed to avoid donor-dependent variability, and total cellular yield and viability were determined using trypan blue exclusion tests. TCSs derived from 6 neonatal piglets were mixed with 4% or 10% HL-60 cancer cells. TCSs without cancer cells were used as controls. TCSs were incubated for 24 h with or without cisplatin (1 µM or 3 µM) or carboplatin (10 µM or 30 µM). The next day, 500,000 cells were recovered and mixed with collagen gel to allow TO generation (n = 15/condition) and cultured for eighteen days on culture inserts. Live imaging to visualize the formation of TOs by density assessment was performed every two to three days. At the end of the culture, TOs were recovered and analyzed by IHC. All experiments were conducted in triplicate.

### 4.2. Animals

Porcine testes (n = 9) for both digestion (n = 3) and TO generation experiments (n = 6) were obtained from a local swine farm. As Belgian legislation allows the castration of piglets for meat production under 21 days of age, we used testes from three- to five-day-old piglets for research. Testes were placed in DMEM/F12 (ThermoFisher Scientific, Ghent, Belgium, 11330032) containing 10 units/mL of penicillin and 10 μg/mL of streptomycin (ThermoFisher Scientific, Ghent, Belgium, 15140122) and were transported within two hours at 4 °C to the lab, where they were immediately decapsulated to remove the epididymis and tunica albuginea.

### 4.3. Tissue Enzymatic Digestion

Testes were minced into fragments of ±1 mm^3^ using surgical blades. Simultaneously, a small fragment was immediately fixated in 4% formaldehyde (VWR Chemicals, Leuven, Belgium, 17A184141) for three hours, embedded in paraffin and characterized based on H&E staining. Only testes showing physiological architecture, and in particular the presence of GCs, were included for the experiments. Testicular tissue fragments were enzymatically digested using five different protocols (E1, E2, E3, E4 and E5, respectively). In brief, testicular fragments were incubated for 10 min at 37 °C in 1.4 mg/mL DNase I (Merck, Overijse, Belgium, 11284932001) further containing 1 mg/mL Collagenase IV (Sigma-Aldrich, Overijse, Belgium, C5138) + 0.5 mg/mL Dispase (ThermoFisher Scientific, Ghent, Belgium, 11510536) (E1), 1 mg/mL Collagenase IV + 1 mg/mL Dispase (E2), 0.5 mg/mL Liberase (Merck, Overijse, Belgium, 5401151001) (E3), 1 mg/mL Liberase (E4) or 1 mg/mL Collagenase IV (E5), respectively. E5, a 2-step enzymatic digestion protocol, was continued with incubation in 0.25% Trypsin/EDTA (ThermoFisher Scientific, Ghent, Belgium, 252000056) + 1.4 mg/mL DNase I for 10 min at 37 °C. At the end of each protocol, the reaction was quenched using phosphate-buffered saline (Lonza, Verviers, Belgium, BE17-516F) containing 10% fetal bovine serum (Sigma-Aldrich, Overijse, Belgium, F7524) and the suspension was filtered through a 70 µm mesh (PluriSelect Life Science, Leipzig, Germany, 43-50070-01). A small fraction of each TCS was used for immediate total cellular yield and viability measurements. The TO generation was conducted with the TCS derived from the enzymatic digestion protocol, resulting in the highest viability and total cellular yield per mg of digested tissue.

### 4.4. Cellular Numbers, Yield and Viability

Trypan blue exclusion testing was used to determine total cellular yield and viability directly after digestion. In brief, 10 µL of trypan blue (Sigma-Aldrich, Overijse, Belgium, T8454) was mixed 1:1 with 10 µL fresh digested TCS and 10 µL of the mixture was added to a 0.0025 mm^2^ Bürker hemocytometer chamber. To allow comparison of the different protocols, cellular yield was normalized per mg of digested testicular tissue. Only viable cells were considered when reporting total cell viability and yields in TCSs. In addition, visual evaluation of digestion progress, looking for remaining tissue pieces in the 70 µm cell strainer at the end of digestion and/or formation of cell debris during cell quantification using trypan blue, was also considered to evaluate digestion protocols.

### 4.5. Artificial Cancer Cell Contamination, Chemotherapy and Culture

The literature revealed that exposure to (0.5 µg/mL) 1.67 µM cisplatin results in similar GC numbers 24 h after exposure, but significantly decreased numbers after 96 h [25]. Therefore, we wanted to evaluate 1 µM cisplatin to verify its gonadotoxicity and 3 µM cisplatin to verify its potential to eradicate cancer contamination. As carboplatin was demonstrated to be around 10× less potent in killing cancer cells compared to cisplatin [21], a 10× higher dose was administered. In total, TCSs from six pig donors were mixed and used to generate TOs. To do so, during a 24 h culture period, 500,000 testicular cells were mixed or not mixed with 4% or 10% human leukemia (HL-60) cancer cells (Sigma-Aldrich, Overijse, Belgium, 98070106) in combination with or without 1 µM cisplatin, 3 µM cisplatin, 10 µM carboplatin or 30 µM carboplatin in DMEM/F12 containing 10% fetal bovine serum, 100 U/mL penicillin and 100 µg/mL streptomycin at 37 °C and 5% CO_2_.

### 4.6. Extracellular Matrix Scaffold Preparation and Culture Conditions for Testicular Organoid Formation

Collagen gel was prepared at the maximum protein concentration obtainable of 2.4 mg/mL by mixing 3 mg/mL Collagen type I (Sopachem, Nazareth, Belgium, 638-00661), DMEM/F12 and reconstitution buffer (Sopachem, Nazareth, Belgium, 635-00791) at a ratio of 8:1:1, on ice to avoid gelation according to manufacturer’s recommendations. To form TOs, cells were recovered after 24 h of culture, centrifuged and resuspended to guarantee homogeneity. After the retrieval of 500,000 cells, they were centrifuged at 400× *g* for 5 min and pelleted into 1.5 mL Eppendorf tubes. By using pipette tips of 200 µL cut at 1 cm from the pointed end, the pellet was recovered in 20 µL of collagen gel mix and placed as a droplet on the lid of a Petri dish. The droplets were incubated for three hours on the inverted Petri dish filled with phosphate-buffered saline (hanging drop). TOs were cultured for eighteen days on 24-well culture inserts (Merck, Overijse, Belgium, PICM01250) in 600 µL of DMEM/F12 containing 10% knockout-serum replacement (ThermoFisher Scientific, Ghent, Belgium, 11509686), 10 ng/mL of fibroblast growth factor 2 (R&D Systems, 233-FB-025), 3 mg/mL of ceftazidime (Biopharma, Italy, Kefadim) and 10 µg/mL gentamycin. Medium was changed every other day and further supplemented with 100 ng/mL bone morphogenetic protein 4 (ThermoFisher Scientific, Ghent, Belgium, PHC9534), 1 µM retinol (Sigma-Aldrich, Overijse, Belgium, R7632), 35 IU/L follicle-stimulating hormone (Merck, Overijse, Belgium, Gonal F) and 2 IU/L human chorionic gonadotropin (Merck, Overijse, Belgium, Ovitrelle) from day nine until the end of the culture.

### 4.7. Live Imaging During TO Culture and Retrieval of TOs

Forming TOs in culture were observed before medium changes every other day with an inverted microscope (Oxion Inverso, EUROMEX, Netherlands) and serial pictures were taken to evaluate the growth of TOs and to select the densest formed TO for further analyses. Density of TOs was evaluated by visual observation of the formation of less light penetrable structures over time as can be seen in Figure 1. Only densely formed TOs were harvested at the end of the eighteen-day culture period and immediately fixed for two hours in 4% formaldehyde prior to embedding in 2% agar and consecutive paraffin embedding. Five-micrometer-thick sections were mounted on Superfrost Plus Slides (VWR Chemicals, Leuven, Belgium, 631-0108) and used for histology and immunohistochemistry.

### 4.8. Histological and Immunohistochemical Analyses

To mitigate the risk of heterogeneous dispersion of testicular cells in the collagen gel, H&E evaluation was performed on the whole TO by random evaluation of each tenth consecutive slide. IHC, using general testicular cell-type-specific markers as demonstrated in the literature, was performed to evaluate the location, numbers and proportions of DDX4^+^ GCs [55], SOX9^+^ SCs [56], CYP11A1^+^ LCs [57] and CD43^+^ HL-60 cancer cells [58] compared to the total number of cells present in TOs. ACTA2^+^ PTMCs [59] were only identified to assess their location as reliable quantification is limited by the difficulty of discerning these cells from each other. In brief, five consecutive sections per TO were deparaffinized in toluene and methanol baths followed by rehydration. Endogenous peroxidase activity was inhibited for 30 min at room temperature in 0.3% hydrogen peroxide, followed by incubation for 50 min at 98 °C in citrate buffer for antigen retrieval. Slides were cooled down for 15 min and washed in Tris-buffered saline (TBS)/Triton 20% (TBST) prior to blocking of non-specific reactions by incubation in TBS containing 10% normal goat serum (Thermofisher Scientific, Ghent, Belgium, 10000C) and 1% bovine serum albumin (Sigma-Aldrich, Overijse, Belgium, A7030) for 30 min. Primary antibodies (Table 5), diluted in TBS containing 1% normal goat serum and 0.1% bovine serum albumin, were added to the slides and incubated overnight at 4 °C. The next day, slides were washed in TBST and incubated with secondary anti-rabbit IgG antibody for one hour at room temperature and washed in TBST. Chromogenic staining using DAB, prepared according to the manufacturer’s instructions (Agilent Technologies, CA, Santa Clara, United States, K3468), was performed at room temperature for 15 min, followed by multiple wash steps. At the end, nuclei were stained using hematoxylin for 20 s at room temperature, and slides were mounted using a Sakura Tissue-Tek mounting device. During all experiments, a positive and negative control omitting the primary antibody was added. Images were acquired by SCAN II (3DHistech, Budapest, Hungary).

### 4.9. Statistical Analyses

Quantification of nuclei and positively labeled cells and statistical analysis were performed using QuPath 0.3.0 software [60]. IHC results are expressed as a proportion indicating the number of cells positive for a cell-type-specific marker in relation to the total number of cells in the TO based on nuclei detection. Data are presented as mean ± SD. Comparisons of cellular proportions and numbers between untreated, 1 µM cisplatin, 3 µM cisplatin, 10 µM carboplatin and 30 µM carboplatin and 0, 4% and 10% initial HL-60 cancer cell contamination were evaluated using a Kruskal–Wallis non-parametric test followed by a post hoc Dunn’s test with Bonferroni correction. Statistical significance was set at α = 0.05 and represented as * *p* ≤ 0.05.

## Figures and Tables

**Table 1:** Total cellular viability and viable cells’ yield normalized per mg of tissue assessed directly after digestion for the 5 enzymatic digestion protocols (E1–E5). Extraction of the cells was identical in all digestion protocols, involving quenching the enzymatic digestion reaction and passing the TCSs through a filter to remove remaining undigested ITT fragments. At the end, all TCSs were centrifuged to remove remaining enzymatic digestion components and resuspended in culture medium. n = 3.

Digestion Protocol	Protocol Details	Viability (%)	Yield (Cells × 10^6^/mg)
E1	1.4 mg/mL DNase I + 1 mg/mL Collagenase IV + 0.5 mg/mL Dispase	95.89 ± 1.84%	0.39 ± 0.20
E2	1.4 mg/mL DNase I + 1 mg/mL Collagenase IV + 1 mg/mL Dispase	95.75 ± 3.40%	0.57 ± 0.33
E3	1.4 mg/mL DNase I + 0.5 mg/mL Liberase	97.73 ± 1.78%	0.84 ± 0.29
E4	1.4 mg/mL DNase I + 1 mg/mL Liberase	97.59 ± 1.13%	0.73 ± 0.32
E5	1st step: 1.4 mg/mL DNase I + 1 mg/mL Collagenase IV 2nd step: 0.25% Trypsin/EDTA + 1.4 mg/mL DNase I	92.74 ± 1.81%	0.73 ± 0.09

**Table 2:** Overview of TO generation efficacy based on the number of selected TOs per initial number of TOs formed. Only densely formed TOs were harvested at the end of the eighteen-day culture period.

	0% HL-60 (n)	4% HL-60 (n)	10% HL-60 (n)
Untreated	80.00% (12)	60.00% (9)	40.00% (6)
1 µM cisplatin	60.00% (9)	60.00% (9)	40.00% (6)
3 µM cisplatin	60.00% (9)	93.33% (14)	80.00% (12)
10 µM carboplatin	60.00% (9)	80.00% (12)	60.00% (9)
30 µM carboplatin	80.00% (12)	60.00% (9)	40.00% (6)

**Table 3:** CD43^+^ quantification expressed as proportions (%) in TOs formed after TCS exposure to chemotherapy and untreated controls normalized per TO and number of recovered TOs (n) for the 2 different HL-60 contamination rates (4% and 10%) at day eighteen.

	4% HL-60 (n)	10% HL-60 (n)
Untreated	36.80 ± 40.93% (9)	37.41 ± 11.70% (6)
1 µM cisplatin	26.40 ± 38.41% (9)	30.44 ± 3.41% (6)
3 µM cisplatin	22.20 ± 34.94% (14)	29.63 ± 29.92% (12)
10 µM carboplatin	18.67 ± 29.72% (12)	32.04 ± 33.56% (9)
30 µM carboplatin	15.51 ± 11.18% (9)	25.16 ± 3.50% (6)

**Table 4:** Overview of DDX4^+^, SOX9^+^, ACTA2^+^ and CYP11A1^+^ cell quantification expressed as proportions compared to the total number of cells in TOs and number of cells in TOs derived from TCSs exposed to chemotherapy and untreated controls grouped for all initial HL-60 contamination conditions normalized per TO. A Kruskal–Wallis non-parametric test followed by a post hoc Dunn’s test with Bonferroni correction was used for comparison of testicular cell numbers among the different treatment groups. * *p* = 0.04.

Treatment Group (n)	DDX4	SOX9	ACTA2	CYP11A1
Untreated (27)	4.02 ± 0.45% * 1242.17 ± 72.65	27.52 ± 10.70% 780.14 ± 41.58	33.97 ± 8.79% 1086.06 ± 261.55	43.38 ± 13.75% 1295.53 ± 309.61
1 µM cisplatin (24)	2.44 ± 0.24% 842.44 ± 214.75	16.52 ± 3.28% 315.99 ± 197.03	22.94 ± 11.90% 475.08 ± 263.34	33.75 ± 18.17% 991.25 ± 283.73
3 µM cisplatin (35)	2.18 ± 0.21% 784.28 ± 427.30	23.80 ± 5.92% 361.27 ± 244.56	20.00 ± 2.94% 540.17 ± 330.96	31.56 ± 10.40% 960.33 ± 654.67
10 µM carboplatin (30)	1.70 ± 0.76% 735.50 ± 116.49	17.46 ± 6.26% 558.11 ± 485.25	21.39 ± 10.82% 563.11 ± 552.84	40.96 ± 7.48% 1358.69 ± 513.80
30 µM carboplatin (27)	1.52 ± 0.36% * 592.17 ± 30.48	19.94 ± 6.24% 389.94 ± 139.72	21.81 ± 6.30% 661.61 ± 108.42	44.01 ± 12.60% 1309.56 ± 956.95

**Table 5:** Antibodies used in this study.

Antibody	Reference	Manufacturer	Dilution	Target Cell	Control Tissue
DDX4	Ab13840	Abcam, Cambridge, UK	1/500	Germ cell	Human adult testis
SOX9	Ab185966	Abcam, Cambridge, UK	1/100	Sertoli cell	Human adult testis
ACTA2	A2547	Sigma-Aldrich, Overijse, Belgium	1/2000	Peritubular myoid cell	Human adult testis
CYP11A1	HPA016436	Sigma-Aldrich, Overijse, Belgium	1/250	Leydig cell	Human adult testis
CD43	1-CD160-05	Quartett, Potsdam, Germany	1/150	HL-60 cancer cell	Human tonsil

## Data Availability

The original contributions presented in this study are included in the article. Further inquiries can be directed to the corresponding author.

## Abbreviations

The following abbreviations were used in this manuscript:

ACTA2	Actin alpha 2
CD43	Cluster of differentiation 43
CYP11A1	Cytochrome P450 Family 11 Subfamily A Member 1
DDX4	DEAD-Box Helicase 4
GC	Germ cell
H&E	Hematoxylin and Eosin
HL-60	Human leukemia 60 cell line
IHC	Immunohistochemistry
ITT	Immature testicular tissue
LC	Leydig cell
PTMC	Peritubular myoid cell
SC	Sertoli cell
SOX9	SRY-box 9
SSC	Spermatogonial stem cell
ST	Seminiferous tubule
TBS	Tris-buffered saline
TBST	TBS/Triton X-100
TCS	Testicular cell suspension
TO	Testicular organoid
